# Memory and Specificity in the Insect Immune System: Current Perspectives and Future Challenges

**DOI:** 10.3389/fimmu.2017.00539

**Published:** 2017-05-09

**Authors:** Dustin Cooper, Ioannis Eleftherianos

**Affiliations:** ^1^Department of Biological Sciences, The George Washington University, Washington, DC, United States

**Keywords:** insects, innate immunity, adaptive immunity, immune priming, immune memory

## Abstract

The immune response of a host to a pathogen is typically described as either innate or adaptive. The innate form of the immune response is conserved across all organisms, including insects. Previous and recent research has focused on the nature of the insect immune system and the results imply that the innate immune response of insects is more robust and specific than previously thought. Priming of the insect innate immune system involves the exposure of insects to dead or a sublethal dose of microbes in order to elicit an initial response. Comparing subsequent infections in primed insects to non-primed individuals indicates that the insect innate immune response may possess some of the qualities of an adaptive immune system. Although some studies demonstrate that the protective effects of priming are due to a “loitering” innate immune response, others have presented more convincing elements of adaptivity. While an immune mechanism capable of producing the same degree of recognition specificity as seen in vertebrates has yet to be discovered in insects, a few interesting cases have been identified and discussed.

## Introduction

Host immune responses against microbial invaders are generally categorized as innate or adaptive. The innate and adaptive immune responses are distinguished by their origin in the host, the way they recognize a microbe or elicitor, and the mechanisms they use to clear or prevent the spread of microbial intruders (1, 2). The adaptive immune response is characterized by two key traits. First, it develops the ability to “remember” a microbe it has encountered before, suggesting that the system has memory, which allows it to respond more quickly during a subsequent infection. Second, it is able to mount a stronger defense, targeted to a particular microbe during a new encounter, suggesting that the defense is specific to that elicitor (3). Further, the pattern-recognition receptors (PRRs) of the adaptive immune response are not found in the host germ line, whereas those associated with the innate immune response are (4). During the initial encounter with a pathogen, somatic recombination in B and T lymphocytes, initiated by recombination-activating genes, gives rise to an adaptive system specifically formed to be able to respond to a secondary infection by the same pathogen (5). The innate immune response is broad, non-specific, and responds similarly against a repeated challenge (6). The PRRs employed by the innate immune system are germ line-encoded and are, therefore, limited in their ability to distinguish between closely related microbes (7). Recognition of pathogen-associated molecular patterns by PRRs allows the system to distinguish between broad classes of microbes and activate a wide array of responses in order to clear the infection (8). Recent research has provided evidence that the innate immune response in insects is more complex and possesses more features than originally thought (9).

## Immune Priming

Immunological priming involves the introduction of dead microbes or a sublethal dose of a live pathogen to the host in order to activate the innate immune response (10, 11). In some insect species, priming confers a strong protective effect against a secondary challenge with an otherwise lethal dose of pathogen. The specificity and duration of the protective effect appears to vary depending on the insect host and type of microbe used as a priming agent (12, 13). In light of evidence pointing toward adaptive aspects in the insect innate immune system, research efforts have mainly focused on the identification of genes or gene clusters that are implicated in the diversity of PRRs required for specificity and memory in a truly adaptive system (14, 15). Identification of a mechanism in insects capable of generating a specific and long-lasting immune response to pathogenic infections would have a major impact on modern immunology. Insects represent a large group of model organisms used to study the molecular and functional basis of the host immune response. Discovery of adaptive immune features in insects would also require that the evolutionary origins of adaptive immunity be revisited.

**Figure 1:**
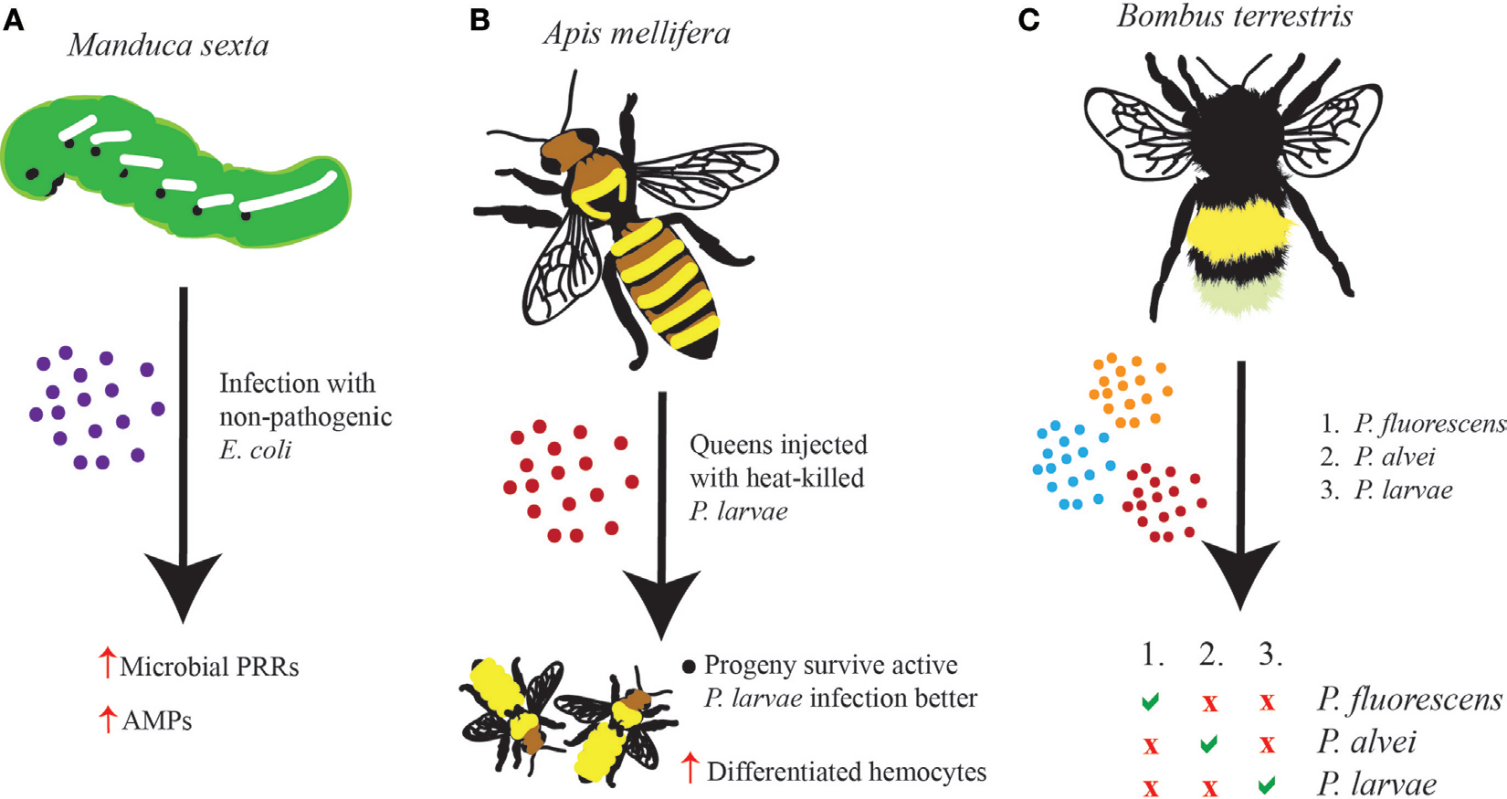
**Evidence of immune priming in insects has emerged in many different forms and to varying extents**. **(A)** Infection of *Manduca sexta* larvae with non-pathogenic *Escherichia coli* leads to the upregulation of microbial pattern-recognition receptors and antimicrobial peptides such that the insect survives better against a secondary infection with a pathogenic microbe, such as *Photorhabdus luminescens*. **(B)** Queen *Apis mellifera* honeybees injected with heat-killed *Paenibacillus larvae* give rise to progeny, which contain as much as three times as many differentiated hemocytes and which survive better against *P. larvae* infection than honeybees whose parents had not been injected. **(C)** The *Bombus terrestris* immune response exhibits a great deal of memory and specificity after being primed with one of three pathogens (*Pseudomonas fluorescens, Paenibacillus alvei*, or *P. larvae*). Survival against a homologous secondary infection is increased; however, no change in survival is seen against heterologous secondary infections.

The insect immune system can be efficiently primed upon exposure to non-pathogenic microbes (16). For example, it has been shown that caterpillars of the tobacco hornworm *Manduca sexta* respond to infection with a non-pathogenic strain of *Escherichia coli* by upregulating an assortment of microbial pattern recognition proteins and antimicrobial peptides (AMPs) (17). This response is persistent enough that upon a second challenge with the virulent insect pathogen *Photorhabdus luminescens*, insect survival is enhanced compared to larvae infected with the pathogenic bacteria, but without being preinfected with *E. coli* (Figure 1A). In addition, the protective effect does persist for at least 48 h post-priming at which point the caterpillars begin preparing to pupate. This demonstrates that the insect innate immune system is functionally able to exhibit long-term non-specific memory-like effect, which, at least in this case, was attributed to the strong antimicrobial activity in the insect hemolymph.

In order to investigate the mechanisms that regulate insect priming, *Parasemia plantaginis* larvae were primed with a non-lethal dose of the pathogenic bacteria *Serratia marcescens*. It was shown that primed larvae contain elevated levels of reactive oxygen species 5 days after oral exposure to the bacteria and just prior to a severe secondary infection (18). These findings indicate that the protective effect against an otherwise lethal septic infection with the same pathogen is probably due to “immunological loitering” rather than an enhanced ability to generate a second immune response. These results were further confirmed by another study that examined priming effects on the greater wax moth *Galleria mellonella* (19). Preinfection experiments revealed that challenging *G. mellonella* larvae with heat-killed *P. luminescens* or *Bacillus thuringiensis* bacteria confers a protective effect by prolonging the survival of insects subsequently infected with either of these pathogens. Interestingly, the priming effect was correlated positively to the priming dose used.

However, the insect innate immune system is not always primed effectively upon microbial preinfection. For example, *Formica selysi* ants infected with a sublethal dose of their natural fungal pathogen *B. bassiana* were examined for priming efficiency (20). Eight to sixteen days after injection of the priming dose, a secondary lethal dose of the fungus was administered. Ants primed with sublethal doses had similar survival rates to the second challenge compared to those that had not been primed, suggesting that no protective effect had been conferred. This result suggests that insect priming is a complex process. Perhaps, in this case, effector molecules produced during priming are not sufficient to provide a protective effect because they are either degraded or depleted by the priming dose. Priming with a smaller dose, or with a different pathogen, may shed more light on the effectiveness of insect priming in this scenario. Another potential answer may suggest a reason why the consistency and persistence of priming is variable among insect species in response to different pathogens. The innate immune response of insects may have evolved a small set of PRRs that are specific to those pathogens that exert the greatest selective pressures on them. While no evidence of this has been presented thus far, such a scenario would provide insects with a very specific immune response when challenged by a pathogen likely to be encountered in nature. Examples of such specificity in the insect innate immune response are outlined in the following sections.

## Transgenerational Immune Priming (TgIP)

One fascinating observation in insect priming involves the transfer of an acquired protective effect from one insect to its offspring. This phenomenon is called TgIP (21). While the mechanisms that regulate this effect have yet to be identified, it is considered a form of innate immune “memory.” In the honeybee *Apis mellifera*, infection with *Paenisbacillus larvae* bacteria is deadly. To test whether a primed response is passed from the queen to the progeny, queens were injected with either heat-killed *P. larvae* or Ringer’s solution as a control, and the ability of the offspring to mount an enhanced immune response was later observed (22). In line with TgIP, offspring whose mother had been primed with *P. larvae* displayed decreased mortality compared to progeny of non-primed honeybees. Further, larval offspring of primed adults contained three times more differentiated hemocytes compared to offspring of non-primed controls. Therefore, it was hypothesized that an unidentified factor is probably transmitted from the primed mother to the egg and is able to then stimulate the differentiation of hemocytes in the honeybee larvae. Interestingly, differentiated hemocytes in honeybees are not directly involved in the elimination of *P. larvae*; instead, they are implicated in the production of AMPs that promote bacterial clearance (Figure 1B). It would be interesting to test whether introducing a different pathogen, either as the priming agent or as the secondary challenge, would also confer a protective effect to the offspring of primed queens.

In the mealworm beetle *Tenebrio molitor*, priming a mother produces TgIP effects in eggs; however, the antimicrobial activity observed does not always correspond to that of the initial priming agent (23). In particular, using zone of inhibition assays, eggs of primed adult female mealworm beetles have been shown to possess elevated antimicrobial activity against Gram-positive bacteria after the mother is primed with either Gram-positive or Gram-negative bacteria. However, priming with fungal pathogens produces weak TgIP effects. The antibacterial activity in eggs may have evolved from the presence of Gram-positive bacteria at various stages during the insect life cycle. If this were the case, it would be interesting to examine TgIP effects in the emerged larvae in response to infection by a range of bacterial pathogens. There is also a possibility that in mealworms, as in honeybees, TgIP modulates effectors that are synthesized during specific insect stages.

Another comprehensive study has demonstrated that TgIP in *M. sexta* can generate different responses depending on the developmental stage of offspring (24). These findings suggest that similar studies examining TgIP effects should always take into account all stages of offspring in both challenged and unchallenged insects. Further, results from this work have indicated that TgIP causes offspring that are not challenged to develop and grow more quickly, suggesting that TgIP probably evolved as a mechanism to protect offspring that are produced in an environment containing pathogens. Enhanced protection would consequently decrease the chances of offspring becoming infected by persistent pathogens (those that *M. sexta* would frequently encounter naturally) and allow them to reach adulthood more rapidly. Crucially, this developmental advantage comes at a cost: adult female offspring of primed parents lay a lower number of eggs, suggesting a trade-off for improved survival. Given the complex interactions involved, research into the genetic mechanisms responsible for inducing these changes will provide a wealth of information, which may be applicable to other systems. It may also shed light on the molecular basis of host–pathogen priming combinations that produce remarkably specific and long-lasting immune responses.

## Memory and Specificity in Insect Immune Priming

Previous studies in insects have demonstrated that immune priming can produce a response specific to the pathogen used to prime the host. Experiments involving priming of the bumblebee *Bombus terrestris* with a Gram-negative (*P. fluorescens*) or two closely related Gram-positive bacteria (*Paenibacillus alvei* and *P. larvae*) and subsequent challenging with either the same bacteria (homologous) or one of the two bacteria with which it had not been primed (heterologous) have shown that primed bees can survive a homologous secondary infection significantly better than a heterologous secondary infection (25). This observation is consistent for all three homologous secondary infections, which readily demonstrates that the insect innate immune system is able to differentiate between two very closely related bacterial species (Figure 1C). These results are distinguished from immunological loitering, first because antibacterial activity only lingered for 14 days postinfection, and second because zone of inhibition assays failed to detect antimicrobial effectors in the hemolymph of the primed insects.

A similar relationship between *Drosophila melanogaster* and *Streptococcus pneumoniae* has also been demonstrated (26). Flies primed with sublethal doses of *S. pneumoniae*, as well as those primed with heat-killed bacteria, display a lifelong ability and remarkable level of specificity in clearing subsequent infections with the same pathogen. Similarly to the bumblebee study, flies are not able to clear *S. pneumoniae* when primed with a related species of bacteria, and protection is not conferred against subsequent infection with related bacteria when flies are primed with *S. pneumonia* pathogens. In an attempt to shed light on the molecular mechanisms underlying this seemingly adaptive response, priming experiments with *D. melanogaster* loss-of-function immune mutants showed that the toll pathway, but not the immune deficiency (Imd) pathway, participates in the primed response of flies to *S. pneumoniae*. In addition, AMPs produced as part of the humoral response are not differentially induced upon a subsequent infection of *S. pneumoniae* in primed flies. Coupled with the observation that *S. pneumoniae* fails to induce the expression of AMPs in *D. melanogaster*, it can be speculated that induction of AMPs most likely is not involved in establishing a primed response. To test the role of cellular immunity in the priming effect, injection of polystyrene beads, which block phagocytosis by plasmatocytes, into unprimed control and *S. pneumoniae* primed flies has produced similar mortality rates between the two experimental conditions. This study pinpoints phagocyte engulfment as a major effector in the secondary response to a pathogen in primed flies.

Both studies have demonstrated that under certain circumstances, the insect innate immune system can be highly specific as well as long lived. These two traits are tenets of the vertebrate adaptive immune response, and their presence in invertebrate organisms suggests that the innate immune response is much more robust than previously thought. While not of the same nature of the vertebrate response, it is now assumed that insects may possess mechanisms capable of generating some adaptive aspects in their immune response.

## A Potential Mechanism for Acquired Immunity in Insects

The vertebrate adaptive immune response is able to respond to nearly any pathogen encountered through the process of somatic recombination. The insect innate immune response, however, has been shown to develop specificity against only a small fraction of pathogenic challenges (27, 28). This observation suggests at least two possibilities for the generation of specific and long-lasting protection in insects. The first possibility is the existence of a set of evolutionarily acquired PRRs capable of mounting a specific response to certain types of pathogens that impose high selective pressure. This possibility would explain recent findings showing that insects activate an adaptive-like immune response, which is specific for certain species of insects and their respective pathogens. It does not, however, provide a mechanism that would support the increased capacity of the insect host immune system after an initial infection. The second possibility is the existence of a mechanism functioning similarly to somatic recombination in vertebrates (13). This mechanism would exhibit diversity and potentially consist of components that are readily induced upon an immune challenge. Such features would allow the system to specifically recognize different immune elicitors without encoding distinct receptors for each one. In addition, the mechanism should be able to readily regulate the activation of immune effectors upon immune recognition. A few candidate molecules with the capacity for these features have been previously identified as possible components of a mechanism by which insects can demonstrate aspects of immune specificity.

**Figure 2:**
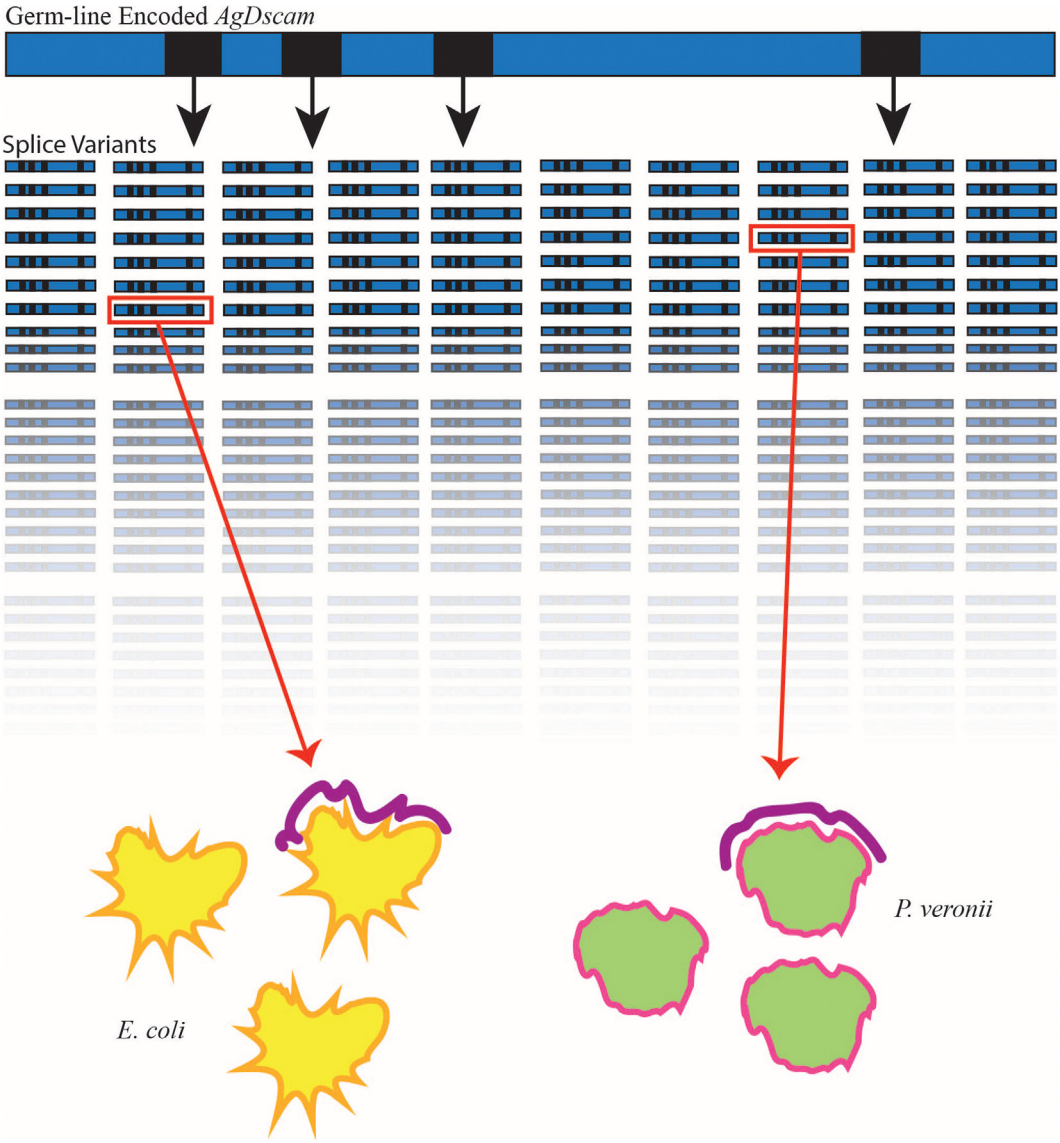
***Anopheles gambiae* Down syndrome cell adhesion molecule (AgDscam), a member of the Ig superfamily, generates semi-specific splice variants in response to various immune elicitors**. In *A. gambiae*, immune elicitors such as *Escherichia coli* (yellow) and *P. veronii* (green) have been shown to lead to the generation of pathogen-specific splice variants (purple) of the germ line-encoded AgDscam. AgDscam (blue bar) contains four exons (black squares), which exhibit alternative splicing, capable of producing 31,920 different isoforms (represented in rows). When mosquitoes are exposed to various bacteria, the repertoire of AgDscam splice variants not only differ but also contain a majority of variants capable of binding to the bacteria (inside red square) to which the insect is exposed.

The Down syndrome cell adhesion molecule (Dscam), a member of the immunoglobulin superfamily, contains four exons, which exhibit alternative splicing. Alternative splicing of these exons produces three hypervariable Ig-domains, resulting in more than 18,000 isoforms in *D. melanogaster*. Further, a variable transmembrane-domain doubles the total number of possible isoforms to a staggering 38,016. These isoforms display different interaction specificity and provide a possible source of diversity for pathogen receptors. The identification of various Dscam isoforms on the surface of immunocompetent cells further supported this theory. Therefore, Dscam is considered a potential candidate molecule for the regulation of adaptive aspects of the insect immune system (29). In *Anopheles gambiae*, Dscam alternative splicing is triggered and controlled by challenge with various immune elicitors, and interfering with Dscam expression affects the phagocytosis and subsequent survival of the mosquitos in response to bacterial infection (30) (Figure 2). Interestingly, no changes in Dscam expression and splicing were found in *D. melanogaster* flies after bacterial infection (31). Therefore, Dscam is an essential component of the innate immune system in some insect species, in which its hypervariability provides the host with a vast collection of pattern recognition molecules.

## Concluding Remarks and Future Questions

Despite prior belief that the insect innate immune response lacks specificity, the expanded use of insects in biomedical research as model organisms has prompted investigation into the intricacies of their response to various types of infection. Previous and recent research has shown that the insect immune response appears much more robust than previously considered. In certain insect species, infection with non-lethal doses of pathogenic bacteria, or priming, confers a protective effect upon subsequent challenge with the same and/or different pathogen. These findings point out the ability of insects to exhibit a form of immune specificity. Further research has suggested that priming of the insect immune system is specific to the insect species and the type of pathogen. The protective effect varies in specificity from providing protection against a wide range of pathogens or specifically against the pathogen to which the insect was initially exposed. In addition, lifelong persistence of immune protection in insects can be accompanied with highly specific recognition of the priming agent.

Previous research has also identified alternative splicing of Dscam in insects as a potential mechanism for generating specific, long-lasting immune responses (32). Hypervariability in Dscam splice isoforms, paired with their expression patterns on the surface of immunocompetent cells and their ability to associate with bacteria (30), suggests a mechanism similar to acquired immunity in vertebrates *via* somatic recombination. While Dscam may not be implicated in promoting adaptive features of the innate immune response in insects, it is profoundly involved in the innate immune response in mosquitoes, but not in flies. Despite these diverse, but certainly exciting, observations in the insect innate immune response, it is evident that the field of insect immunology is much more complex than previously envisioned. A great deal of the priming effect in insects and its impact on certain immune functions remains currently unexplored; therefore, future research using insect models promises a generation of thrilling information that will potentially uncover the relationship between immune priming and physiological responses in insects.

## Author Contributions

DC wrote the paper and IE revised it.

## Conflict of Interest Statement

The authors declare that the research was conducted in the absence of any commercial or financial relationships that could be construed as a potential conflict of interest.
